# Advancing measurement-based care through triangle of care: Development and feasibility of the Transdiagnostic Global Impression – Psychopathology scale for patients and informants

**DOI:** 10.1192/j.eurpsy.2025.10069

**Published:** 2025-08-05

**Authors:** Roger S. McIntyre, Zsofia Borbala Dombi, Agota Barabassy, Thomas Brevig, György Németh, Christoph U. Correll

**Affiliations:** 1 Department of Psychiatry, University of Toronto, Toronto, ON, Canada; 2 Department of Psychiatry, University of Oxford, Oxford, UK; 3 Global Medical Division, Gedeon Richter Plc., Budapest, Hungary; 4 Department of Psychiatry, The Zucker Hillside Hospital, Northwell Health, Glen Oaks, NY, USA; 5 Department of Psychiatry and Molecular Medicine, Donald and Barbara Zucker School of Medicine at Hofstra/Northwell, Hempstead, NY, USA; 6 Department of Child and Adolescent Psychiatry, Charité Universitätsmedizin Berlin, Berlin, Germany; 7 German Center for Mental Health (DZPG), partner site Berlin, Berlin, Germany; 8 Center for Psychiatric Neuroscience, The Feinstein Institute for Medical Research, Northwell Health, New Hyde Park, NY, USA

**Keywords:** measurement-based care, psychiatric scale, psychopathology, transdiagnostic assessment tool, triangle of care

## Abstract

**Background:**

Measurement-based care (MBC) is widely recommended in psychiatry but remains underutilized in routine clinical settings. The Transdiagnostic Global Impression – Psychopathology (TGI-P) scale was developed to provide a brief yet comprehensive assessment of 10 core transdiagnostic symptom domains. To support more inclusive care and promote patient and caregiver engagement in treatment planning, two new versions of the TGI-P, that is, a patient-rated and a separate informant-rated, were developed, complementing the previously published clinician-rated version.

**Methods:**

The patient and informant versions mirror the original clinician-rated TGI-P, assessing the identical 10 domains using a seven-point Likert severity scale, with results displayed via a personalized symptom map. A user satisfaction/feasibility study was conducted with 50 participants (25 patients and 25 caregivers) from the UK and US. After completing the scale, participants provided feedback on its clarity, usability, emotional impact, and comparative utility.

**Results:**

Most participants completed the scale in less than 5 min. Instructions were considered clear, and the format was rated easy to follow. Response options were deemed appropriate by 86% of participants, and the visual output was widely appreciated. While one-third reported mild emotional triggering, overall burden was described as manageable. Approximately, three-quarters of participants rated the TGI-P as equal to or better than other tools they had used.

**Conclusions:**

TGI-P patient and informant versions were developed and, informed by the feasibility study, refined to offer brief, user-friendly tools that support multi-informant assessment as input to MBC. Both versions of the TGI-P, with their graphical output, may support shared understanding and collaborative decision making among clinicians, patients, and caregivers. A validation study of the TGI-P is underway.

## Highlights

- The Transdiagnostic Global Impression – Psychopathology scale (TGI-P) assesses and visualizes severity across 10 transdiagnostic psychiatric symptom domains.
- Both patient and informant versions of the TGI-P scale were completed in under 5 min by most participants, with high ratings for clarity and ease of use.
- The new TGI-P versions enable multi-informant assessment and were preferred over other tools by most participants, highlighting their potential to enhance collaborative and measurement-based care.

## Introduction

Measurement-based care (MBC), the continuous administration of validated rating scales in everyday practice [1, 2], is a cornerstone of effective psychiatric practice [2–5]. MBC has been shown to improve consistency, appropriateness, quality, and cost effectiveness of treatment in depression [5, 6]. Nevertheless, the use of measurement-based devices in clinical settings in uncommon, contributing to heterogeneity of care and reducing the likelihood of optimized outcomes [7–9]. To help address this gap, the Transdiagnostic Global Impression – Psychopathology (TGI-P) scale was developed as the first detailed and multidimensional transdiagnostic symptom scale in psychiatry, assessing 10 core symptom domains (positive, negative, manic, depressive, hostility, self-harm, addiction, cognitive, anxiety and sleep symptoms) using a simple 1–7 severity rating, modelled on the Clinical Global Impression – Severity (CGI-S) scale [10]. The TGI-P is designed to be concise, intuitive, and clinically useful; facilitating rapid assessment and visual feedback through a symptom map that enables the global understanding of psychopathology and of its evolution over time [10].

The importance of including multiple perspectives in psychiatric evaluation is increasingly recognized [11, 12]. The “Triangle of Care” (ToC) model highlights the value of integrating views from clinicians, patients, and caregivers to gain a more complete picture of mental health conditions [13]. This process is fundamental for triadic shared decision making [14], as patient and caregiver insights not only help contextualize symptoms but also contribute to treatment planning and the building of a therapeutic alliance [15].

Consequently, patient-reported outcome measures (PROMs) have gained attention and are increasingly utilized [16], providing valuable insights and reflecting a shift toward more patient-centred approaches [17, 18]. While offering unique insights, PROMS can also be adversely influenced by non-response, fatigue, timing, language, and recall biases [16]; as well as may introduce potential biases associated with subjective self-assessment, especially in symptom domains where insight may be compromised, such as mania or psychosis [19]. Nonetheless, research shows moderate-to-strong correlations between patient/caregiver reports and clinician ratings, supporting the value of integrating these different, yet complementary perspectives [18, 20]. Importantly, PROMs reflect internal experiences, such as mood changes and subjective distress better than external observations, and such internal states are not necessarily explored in clinical interviews [21]. However, there are several barriers to implementing PROMs, including overly complex formats and content, as well as the time involved in completing the self-reports [22]. Indeed, measures that are “quick,” “simple,” and “well-explained” are much preferred [22].

Against this background, patient- and informant-rated versions of the TGI-P scale were developed which had been introduced as a clinician-rated instrument first [10]. This paper outlines the development process and presents findings from an initial feasibility study of the TGI-P patient and informant versions.

## Methods

### The development of the TGI-P clinician version

The 10 symptom domains included in the TGI-P were originally defined based on a synthesis of expert consensus and empirical research, as outlined in our previously published clinician-scale paper [10]. Each symptom domain of the TGI-P is rated on a seven-point severity scale (1 – normal/not at all; 2 – minimal; 3 – mild; 4 – moderate; 5 – marked; 6 – severe; 7 – extreme), which is aligned with the widely accepted and used CGI-S scale [10]. The ratings are then plotted onto a symptom map, creating a visual clinical footprint that aids in understanding the patient’s current mental health status [10]. The development process followed key principles of scale design as summarized by Boateng et al., including domain conceptualization, item generation, and cognitive testing [23].

### The development of the TGI-P patient and informant version

The primary aim in developing the patient and informant versions of the scale was to produce two formats that mirrored the original clinician-rated version while remaining easily understandable to non-clinician users. To achieve this goal, each symptom domain was reformulated as a question, using non-clinical language to enhance accessibility (e.g., hallucinations from the positive symptom domain were described as “seeing or hearing things that others could not”). At the same time, it was essential to retain the same response format as the original scale to ensure consistency across versions.

In the finalized versions of the TGI-P patient and informant scales, all patient items begin with the prompt: *“In the past week, to what extent have you…,”* while the informant version uses: *“In the past week, how much have you noticed that the person you provide care for….”* The initial item formulations were drafted by Zs.B.D. and subsequently reviewed and refined collaboratively with the co-authors to ensure clarity, clinical relevance, and accessibility across user groups. Each version is accompanied by a brief introductory text designed to orient respondents to the purpose and format of the scale. For patients, the instruction reads: *“The Transdiagnostic Global Impression – Psychopathology (TGI-P) scale aims to gain a deeper understanding of the symptoms you have experienced and their intensity over the past 7 days. The scale uses a 7-point rating from 1 (not at all) to 7 (extreme severity). Please read each question and circle the number that best corresponds to your experience. If you did not experience a certain symptom at all, please circle 1 (not at all).”* For informants, the adapted instruction states: *“The Transdiagnostic Global Impression – Psychopathology (TGI-P) scale aims to gain a deeper understanding of the symptoms experienced by the person you care for over the past 7 days. Please read each question carefully and circle the number that best reflects your impression of the intensity of their symptoms. If they did not experience a certain symptom at all, please circle 1 (not at all).”* These introductions were designed to improve user comprehension and support more consistent, informed responses.

### Survey design

In line with established scale development procedures, the next step after item formulation was to administer the scale to a sample representative of the target population to assess feasibility and user satisfaction [23]. To this end, a targeted survey was conducted in collaboration with a research agency (Inspira Research). A total of 50 participants (25 patients and 25 informants) were recruited from the UK and US and their identities remained anonymous to the authors. Inclusion criteria for patients were a self-reported diagnosis of bipolar disorder, major depressive disorder, or schizophrenia. Informants were eligible if they provided care for someone with one of these diagnoses. Notably, informants were not matched with the patients.

Participants completed the draft TGI-P scales along with a structured feedback questionnaire. The assessment focused on four key areas: (1) clarity and comprehensibility of the wording and instructions; (2) usability and emotional impact; (3) appropriateness of the response options; and (4) perceived comparability to other commonly used rating tools. Additionally, open-ended questions invited suggestions for improving the format and design of the scale. Importantly, responses to the TGI-P scale items themselves were not recorded; only feedback about the questionnaire was collected and analysed.

## Results

### Participant characteristics

A total of 50 participants took part in the study, comprising 25 patients and 25 caregivers. Of the patients, 12 were from the UK and 13 from the US. Among caregivers, 13 were based in the UK and 12 in the US (Table 1). The majority of participants were female (72%), with identical gender distributions among patients and caregivers. Participants ranged in age from 18 to over 60, with a mean age of 44.5 years (SD = 9.9). Nearly half of the sample (46%) were between 18 and 39 years old, and smaller proportions were aged 40–49 years (26%), 50–59 years (20%), and 60 years or older (8%). Caregivers were more likely to fall into the youngest age group (52%), while patients were more evenly split between 18–39 and 40–49 years (40% each).

**Table 1.** Participant characteristics of the patient-caregiver survey

	Total, *n* = 50	Patients, *n* = 25	Caregivers, *n* = 25
Country, *n* (%)			
From UK	25 (50.0)	12 (48.0)	13 (52.0)
From US	25 (50.0)	13 (52.0)	12 (48.0)
Sex, *n* (%)			
Male	14 (28.0)	7 (28.0)	7 (28.0)
Female	36 (72.0)	18 (72.0)	18 (72.0)
Age groups, *n* (%)			
18–39 years	23 (46.0)	10 (40.0)	13 (52.0)
40–49 years	13 (26.0)	10 (40.0)	3 (12.0)
50–59 years	10 (20.0)	3 (12.0)	7 (28.0)
60+	4 (8.0)	2 (8.0)	2 (8.0)
Age, mean (SD)	44.0 (10.2)	43.9 (10.1)	42.4 (11.4)
Education, *n* (%)			
High school	12 (24.0)	5 (20.00)	7 (28.0)
Vocational school	4 (8.0)	2 (8.0)	2 (8.0)
University	34 (68.0)	18 (72.0)	16 (64.0)
Household size, *n* (%)			
1	11 (22.0)	9 (36.0)	2 (8.0)
2	13 (26.0)	6 (24.0)	7 (28.0)
3	11 (22.0)	2 (8.0)	9 (36.0)
4+	15 (30.0)	8 (32.0)	7 (28.0)
Diagnosis of patients/patients whom caregivers provide care for, *n* (%)			
Bipolar disorder	17 (34.0)	9 (36.0)	8 (32.0)
Major depression	16 (32.0)	10 (40.0)	6 (24.0)
Schizophrenia	17 (34.0)	6 (24.0)	11 (44.0)

Educational attainment was generally high, with 68% of participants reporting a university degree, including 72% of patients and 64% of caregivers. Smaller proportions reported completing high school only (24%) or vocational training (8%). Household sizes varied: 30% of participants lived in households of four or more, 26% lived with one other person, 22% with two others, and 22% lived alone. Notably, a higher proportion of patients (36%) lived alone compared to caregivers (8%), while caregivers were more likely to live in three-person households (36% vs. 8% of patients).

Regarding clinical characteristics, among the patient group, major depressive disorder was the most frequently reported diagnosis (40%), followed by bipolar disorder (36%) and schizophrenia (24%). Caregivers reported providing support most often to individuals with schizophrenia (44%), followed by bipolar disorder (32%) and major depression (24%).

### Opinion on rating scales in general

Participants varied in their experience and attitudes toward using rating scales (Table 2). While only a small proportion (8%) reported completing rating scales frequently or on a weekly basis, 44% did so occasionally (monthly), and 36% reported rarely completing them (less than monthly). A minority (12%) stated they had never used rating scales. Interestingly, caregivers were more likely than patients to report occasional use (56% vs. 32%), whereas patients more frequently reported rare use (48% vs. 24%).

**Table 2.** General attitudes towards rating scales

	Total, *n* = 50	Patients, *n* = 25	Caregivers, *n* = 25
Frequency of filling out rating scales, *n* (%)			
Frequently/weekly	4 (8)	2 (8)	2 (8)
Occasionally/monthly	22 (44)	8 (32)	14 (56)
Rarely/less than monthly	18 (36)	12 (48)	6 (24)
Never	6 (12)	3 (12)	3 (12)
Helpfulness of rating scales, *n* (%)			
Very helpful	13 (26)	5 (20)	8 (32)
Somewhat helpful	17 (34)	10 (40)	7 (28)
Neutral	14 (28)	6 (24)	8 (32)
Somewhat exhausting	4 (8)	2 (8)	2 (8)
Very exhausting	2 (4)	2 (8)	0 (0)
Preference for visual output, *n* (%)			
Much more preferred	29 (58)	14 (56)	15 (60)
Little more preferred	14 (28)	8 (32)	6 (24)
Not preferred	7 (14)	3 (12)	4 (16)
Format of scale, *n* (%)			
Digital	29 (58)	13 (52)	16 (64)
Paper	6 (12)	3 (12)	3 (12)
Both	15 (30)	9 (36)	6 (24)

Perceived helpfulness of rating scales was generally positive. Overall, 26% of participants found them very helpful and 34% somewhat helpful, with patients more likely to report “somewhat helpful” (40%) compared to caregivers (28%). About 28% of the total sample felt neutral, and a small minority found rating scales somewhat (8%) or very exhausting (4%).

A clear preference for visual outputs from rating tools was evident, with 58% of participants indicating they “much more preferred” visual formats and an additional 28% reporting a “little more” preference. Only 14% reported no preference for visual output. Format preferences were also explored: 58% preferred digital formats, 12% preferred paper, and 30% liked both. Caregivers were slightly more likely to favour digital formats (64%) compared to patients (52%).

### Opinion on the TGI-P scale

Participant feedback on the TGI-P scale is provided in Table 3. The majority (68%) reported that the aim of the scale was very clear, with the remaining 32% finding it somewhat clear; no participant reported confusion about the purpose. Instructions were generally easy to follow; 80% found them very easy, and another 16% rated them as easy. Confidence when completing the scale was high: 76% felt completely confident, and the remaining 24% felt somewhat confident. No participant reported needing assistance. Most participants (72%) completed the scale in under 5 min, with nearly all others completing it within 5–10 min. The process of completing the scale was described as very easy or easy by 94% of participants, with no reports of difficulty.

**Table 3.** Results of the patient-caregiver survey

Opinion about the TGI-P scale, *N* (%)	Total, *n* = 50	Patients, *n* = 25		Caregivers, *n* = 25	
		UK *n* = 12	US *n* = 13	UK *n* = 13	US *n* = 12
Understandability of aim					
Yes, very clear	34 (68)	7 (58)	7 (54)	13 (100)	7 (58)
Somewhat clear	16 (32)	5 (42)	6 (46)	0 (0)	5 (42)
Not clear	0 (0)	0 (0)	0 (0)	0 (0)	0 (0)
Understandability of instructions					
Very easy to follow	40 (80)	10 (83)	11 (85)	13 (100)	6 (50)
Easy to follow	8 (16)	2 (17)	2 (15)	0 (0)	4 (33)
Neutral	2 (4)	0 (0)	0 (0)	0 (0)	2 (17)
Confusing	1 (2)	0 (0)	0 (0)	0 (0)	0 (0)
Level of confidence when filling the scale					
Yes, completely	38 (76)	7 (58)	11 (85)	12 (92)	8 (67)
Somewhat	12 (24)	5 (42)	2 (15)	1 (8)	4 (33)
No, I needed help	0 (0)	0 (0)	0 (0)	0 (0)	0 (0)
Length of filling out					
Less than 5 min	36 (72)	10 (83)	9 (69)	8 (62)	9 (75)
5–10 min	13 (26)	2 (17)	4 (31)	5 (38)	2 (17)
More than 10 min	1 (2)	0 (0)	0 (0)	0 (0)	1 (8)
Easiness of filling out					
Very easy	31 (62)	6 (50)	8 (62)	11 (85)	6 (50)
Easy	16 (32)	5 (42)	5 (38)	1 (8)	5 (42)
Neutral	3 (6)	1 (8)	0 (0)	1 (8)	1 (8)
Difficult	0 (0)	0 (0)	0 (0)	0 (0)	0 (0)
Very difficult	0 (0)	0 (0)	0 (0)	0 (0)	0 (0)
Difficulties during filling out					
No difficulties	48 (96)	11 (91)	13 (100)	13 (100)	11 (91)
A few minor difficulties	2 (4)	1 (8)	0 (0)	0 (0)	1 (8)
Many difficulties	0 (0)	0 (0)	0 (0)	0 (0)	0 (0)
Understandability of wording^a^					
Question 1 – Positive symptoms	3 (6)	1 (8)	0 (0)	2 (15)	0 (0)
Question 2 – Hostility symptoms	1 (2)	0 (0)	0 (0)	1 (8)	0 (0)
Question 3 – Manic symptoms	4 (8)	2 (17)	0 (0)	2 (15)	0 (0)
Question 4 – Addiction symptoms	3 (6)	1 (8)	0 (0)	1 (8)	1 (8)
Question 5 – Sleep symptoms	1 (2)	0 (0)	0 (0)	1 (8)	0 (0)
Question 6 – Negative symptoms	1 (2)	0 (0)	0 (0)	1 (8)	0 (0)
Question 7 – Self-harm symptoms	5 (10)	1 (8)	1 (8)	2 (15)	1 (8)
Question 8 – Depressive symptoms	1 (2)	0 (0)	0 (0)	1 (8)	0 (0)
Question 9 – Cognitive symptoms	1 (2)	0 (0)	0 (0)	1 (8)	0 (0)
Question 10 – Anxiety symptoms	1 (2)	0 (0)	0 (0)	1 (8)	0 (0)
Evaluation of scale response options					
Very appropriate	43 (86)	9 (75)	13 (100)	11 (85)	10 (83)
Somewhat appropriate	7 (14)	3 (25)	0 (0)	2 (15)	2 (17)
Not appropriate	0 (0)	0 (0)	0 (0)	0 (0)	0 (0)
Evaluation of visual output					
Very helpful	29 (58)	6 (50)	7 (54)	10 (77)	6 (50)
Somewhat helpful	20 (40)	6 (50)	6 (46)	3 (23)	5 (42)
Not helpful	1 (2)	0 (0)	0 (0)	0 (0)	1 (8)
Overwhelmingness					
Not at all overwhelmed	37 (74)	8 (67)	11 (85)	10 (77)	8 (67)
Slightly overwhelmed	13 (26)	4 (33)	2 (15)	3 (23)	4 (33)
Very overwhelmed	0 (0)	0 (0)	0 (0)	0 (0)	0 (0)
Burden					
Not at all burdensome	43 (86)	10 (83)	12 (92)	12 (92)	9 (75)
Slightly burdensome	7 (14)	2 (17)	1 (8)	1 (8)	3 (25)
Very burdensome	0 (0)	0 (0)	0 (0)	0 (0)	0 (0)
Emotional trigger					
Not at all triggered	34 (68)	7 (58)	9 (69)	10 (77)	8 (67)
Slightly triggered	15 (30)	4 (33)	4 (31)	3 (23)	4 (33)
Very triggered	1 (2)	1 (8)	0 (0)	0 (0)	0 (0)
Comparison to other scales					
Much better	16 (32)	2 (17)	2 (15)	9 (69)	3 (25)
Better	22 (44)	7 (58)	6 (46)	4 (31)	5 (42)
About the same	11 (22)	3 (25)	5 (38)	0 (0)	3 (25)
Worse	1 (2)	0 (0)	0 (0)	0 (0)	1 (8)
Much worse	0 (0)	0 (0)	0 (0)	0 (0)	0 (0)
Future improvements					
Shorter formats	25 (50)	4 (33)	4 (31)	9 (69)	8 (67)
Simpler wording	18 (36)	6 (50)	3 (23)	7 (54)	2 (17)
Clearer instructions	9 (18)	4 (33)	1 (8)	2 (15)	2 (17)
Other	2 (4)	0 (0)	1 (8)	0 (0)	1 (8)

a only ‘No’ answers displayed.

Only minor wording aspects were noted, primarily relating to items on self-harm (10%), manic symptoms (8%), and positive or addiction symptoms (6% each), mostly among UK caregivers. Despite these few points, the response options were deemed very appropriate by 86% of participants, with no one finding them inappropriate. Most participants (96%) experienced no difficulties during completion. Regarding the scale’s visual output, a graphical symptom profile, 58% found it very helpful, and 40% somewhat helpful.

Most participants (74%) reported not feeling overwhelmed by the TGI-P scale, with the highest comfort observed among UK caregivers (85%) and UK patients (77%). A quarter of respondents felt slightly overwhelmed, but no participants across any group reported feeling very overwhelmed. The tool was generally not perceived as burdensome, with 86% rating it as not at all burdensome and no one reporting it as very burdensome. Emotional triggering was minimal overall; 68% were not triggered, 30% slightly triggered, and only one patient (8%) in the UK group reported being very triggered.

When compared to other clinical rating tools, 76% of participants rated the TGI-P as better or much better, with only one person describing it as worse. Suggestions for future improvements included further shortening the format (50%), simplifying wording (36%), and improving clarity of instructions (18%). The foregoing suggestions were more frequent among UK caregivers and US patients.

In response to the final open-ended question, 68% of participants voluntarily provided additional comments. Of these, 26% noted that the scale was clear and easy to understand, 30% found it helpful and elaborated on its usefulness, and 12% identified challenges or proposed suggestions for further refinement. While the visual output and overall wording were generally well received, several participants recommended simplifying certain terms and including explanatory notes alongside the visual feedback to explain its nature and purpose better. A small number of respondents also reflected on the concept itself, noting that completing the scale could be emotionally triggering.

### Finalisation of the TGI-P scale patient and informant versions

Following analysis of participant feedback from the survey, the patient and informant versions of the TGI-P scale were finalized through targeted refinements. While the total number of items remained unchanged, several questions were reworded to improve clarity, address commonly reported challenges, and reduce perceived complexity. Notably, the phrasing of each item was adjusted to begin unanimously for patients with: *“In the past week, how severe were your symptoms/experiences of…,”* and for informants with: *“In the past week, how severe were the symptoms/experiences of the person you provide care for in terms of….”* These revisions aimed to anchor responses more clearly within a defined time frame and to improve interpretability across both TGI-P user groups. The refinement was a collaborative work involving all members of the development team. Details of wording changes are in the Supplementary Material.

In response to concerns about the clarity of the visual output, the introductory texts were also revised to better explain the scale’s purpose and how results are used. For patients, the updated instruction now reads: *“The TGI-P scale is designed to provide a global overview of your symptoms and mental health experiences over the past 7 days. Please read each question carefully and rate how severe these symptoms and/or experiences have been for you on average during the past week, using a scale from 1 (not at all/normal) to 7 (extreme). If you did not experience a particular symptom, please select 1. Your responses will be plotted onto a symptom map, creating a clinical footprint of your current state – helping your care team better understand your overall symptom profile and areas that may need attention.”*

For informants, the corresponding version states: *“The TGI-P scale is designed to provide a global overview of the symptoms and mental health experiences of the one you provide care for over the past 7 days. Please read each question carefully and rate how severe these symptoms and/or experiences have been for the one you provide care for on average during the past week, using a scale from 1 (not at all/normal) to 7 (extreme). If they did not experience a particular symptom, please select 1. Your responses will be plotted onto a symptom map, creating a clinical footprint of their current state – helping the care team better understand their overall symptom profile and areas that may need attention.”*

The final pen-and-paper versions of the TGI-P patient and informant scales are in the Supplement.

## Discussion

### Summary of key findings

The findings based on the user satisfaction/feasibility study support the usability, acceptability, and clarity of the TGI-P patient and informant scales. Most participants found the tool quick and easy to complete, with clear instructions and appropriate response options. The visual symptom map was well received, and the scale was generally preferred over traditional tools. Presenting the symptom composition, as opposed to using a composite score (which is a common method for summarizing scale measurements), may allow clinicians to describe individual symptoms more clearly to patients and caregivers. This approach is especially relevant when the TG-P is filled out regularly and, therefore, symptom evolution is tracked via the change of the symptom map; the closer the shape moves to the middle, the less severe the symptoms are. Results of the survey also support this by showing that patients and caregivers have a clear preference for visual output.

The findings suggest that the TGI-P scale is broadly acceptable in terms of emotional and cognitive load, though slight emotional responses and perceived burden among some participants warrant attention in future implementations. Indeed, one third of the patients felt slightly triggered when completing the scale, which might require attention, particularly in case of vulnerable individuals. All in all, the results suggest preliminary feasibility of using the scale in everyday practice.

### The role of TGI-P in advancing measurement-based care through triangle of care

The present findings align with growing interest in incorporating patient and informant perspectives into psychiatric care [24]. Previous research highlights the value of multi-informant ratings, particularly in chronic mental illness, where insight and symptom variability may impact clinician-only assessments [17, 18]. Patient- and informant-reported tools can capture subjective distress, identify unmet needs, and improve engagement in care [21]. However, common concerns with these tools include complexity, emotional burden, and questionable alignment with clinician assessments [22]. The TGI-P patient and informant versions address many of these limitations by being brief and intuitive. Additionally, the visual output of the TGI-P, a symptom map, provides a novel way to describe clinical states and also an opportunity to discuss areas that need further support with patients and informants openly. By comparing the three versions of the TGI-P, with just one look, clinicians have the ability to get a comprehensive picture of the symptom complex the patient experiences from multiple perspectives, which can also be tracked over time and put into the context of treatment changes.

### Limitations

Several limitations must be acknowledged. First of all, the present study was a user satisfaction/feasibility study, which cannot provide information on validity and reliability. Therefore, proper psychometric testing is underway, focusing on reliability, construct validity, and inter-rater agreement with clinician-rated versions. Second, the survey sample was relatively small and limited to the UK and US. Third, the high average educational attainment may have positively biased results regarding scale clarity and usability. Education is an important factor when analysing feasibility and user satisfaction as higher educational attainment may increase the likelihood of favourable results. Fourth, patients and caregivers were not matched, so that results could not be compared. Fifth, psychiatric diagnoses were self-reported, and no external verification was obtained. Finally, the TGI-P scales are symptom-focused. However, to provide a broader, transdiagnostic assessment platform of meaningful clinical outcomes that can inform care, several additional TGI scales are being developed concurrently. These include the TGI – Adverse Events, TGI – Functioning, TGI – Caregiver burden, TGI – Satisfaction with Life, and TGI – Satisfaction with Care scales. Except for the TGI Satisfaction with Life and TGI Satisfaction with Care scales that are patient-rated only, all other TGI scales are developed as a clinician, patient and informant version.

### Future research

Future research is needed to establish the validity and reliability of the TGI-P scales. Such studies will need to include large and diverse populations, and test the implementation of the TGI-P Patient and/or TGI-P Informant versions across different treatment settings. To this end, psychometric validation of the TGI-P patient and informant versions together with the TGI-P Clinician version is currently ongoing, including assessment of reliability, construct validity, and inter-rater agreement with clinician-rated versions. Longitudinal data collection will further help evaluate the scale’s sensitivity to change and its utility in routine monitoring as part of clinical care. In addition, the impact on overall satisfaction with care and cost effectiveness of treatment when routinely implementing TGI-P is a future research consideration [25].

## Conclusion

The TGI-P patient and informant scales were developed to offer a brief, transdiagnostic tool suitable for routine clinical use. Designed to complement the clinician-rated version, these two companion scales empower patients and informants to contribute to the understanding of symptom severity across 10 domains. The visual output of the TGI-P may support shared understanding and decision-making between clinicians, patients, and families. With proper validation, the TGI-P scales (alone or together with the additional TGI scales that are being developed) are hoped to help bridge the gap between time-consuming measurement-based research data collection in selected patient samples and scalable and informative MBC implemented as part of real-world psychiatric practice.

## Supporting information

10.1192/j.eurpsy.2025.10069.sm001 McIntyre et al. supplementary material McIntyre et al. supplementary material

## Data Availability

The data that support the findings of this study are available from the corresponding author upon reasonable request.

## Supplementary material

The supplementary material for this article can be found at http://doi.org/10.1192/j.eurpsy.2025.10069.

## References

[1] McIntyre RS. Using measurement strategies to identify and monitor residual symptoms. J Clin Psychiatry. 2013;74:14–8. 10.4088/JCP.12084su1c.03. 24191973

[2] Dunlop BW, Gray J, Rapaport MH. Transdiagnostic clinical global impression scoring for routine clinical settings. Behav Sci. 2017;7:40. 10.3390/bs7030040. 28653978 PMC5618048

[3] Guo T, Xiang YT, Xiao L, Hu CQ, Chiu HFK, Ungvari GS, et al. Measurement-based care versus standard care for major depression: a randomized controlled trial with blind raters. Am J Psychiatry. 2015;172:1004–13. 10.1176/appi.ajp.2015.14050652. 26315978

[4] Bauer M, Pfennig A, Linden M, Smolka MN, Neu P, Adli M. Efficacy of an algorithm-guided treatment compared with treatment as usual: a randomized, controlled study of inpatients with depression. J Clin Psychopharmacol. 2009;29:327–33. 10.1097/JCP.0b013e3181ac4839. 19593170

[5] Maj M, Stein DJ, Parker G, Zimmerman M, Fava GA, De Hert M, et al. The clinical characterization of the adult patient with depression aimed at personalization of management. World Psychiatry 2020;19:269–93. 10.1002/wps.20771. 32931110 PMC7491646

[6] McIntyre RS, Lee Y, Mansur RB. Treating to target in major depressive disorder: response to remission to functional recovery. CNS Spectr. 2015;20:17–31. 10.1017/S1092852915000826. 26683526

[7] Correll CU, Kishimoto T, Nielsen J, Kane JM. Quantifying clinical relevance in the treatment of schizophrenia. Clin Ther. 2011;33:B16–. 39. 10.1016/j.clinthera.2011.11.016. 22177377 PMC3298768

[8] Zimmerman M, McGlinchey JB. Why don’t psychiatrists use scales to measure outcome when treating depressed patients? J Clin Psychiatry. 2008;69:1916–19. 10.4088/JCP.v69n1209. 19192467

[9] Hatfield DR, Ogles BM. Why some clinicians use outcome measures and others do not. Admin Policy Mental Health Mental Health Serv Res. 2007;34:283–91. 10.1007/s10488-006-0110-y. 17211715

[10] Correll CU, Dombi ZB, Barabássy Á, Németh G, Brevig T, McIntyre RS. The Transdiagnostic global impression – psychopathology scale (TGI-P): initial development of a novel transdiagnostic tool for assessing, tracking, and visualising psychiatric symptom severity in everyday practice. Eur Neuropsychopharmacol 2024;88:31–9. 10.1016/j.euroneuro.2024.07.012. 39121713

[11] Smith MB. The importance of multiple perspectives in psychiatry. Psychol Psychother Res Stud. 2018;1:3. 10.31031/PPRS.2018.01.000511.

[12] Peters ME, Taylor J, Lyketsos CG, Chisolm MS. Beyond the DSM: the perspectives of psychiatry approach to patients. Prim Care Compan CNS Disord. 2012;14(1):PCC.11m01233. 10.4088/PCC.11m01233. PMC3357579 22690367

[13] The Carers Trust. The triangle of care, carers included: a guide to best practice in mental health care 2013. https://www.crisiscareconcordat.org.uk/inspiration/the-carers-trust-thetriangle-of-care-carers-included-a-guide-to-best-practice-in-mentalhealth-care/. Accessed 30 Apr 2025.

[14] Schuster F, Holzhüter F, Heres S, Hamann J. ‘Triadic’ shared decision making in mental health: Experiences and expectations of service users, caregivers and clinicians in Germany. Health Expect 2021;24:507–15. 10.1111/hex.13192. 33450125 PMC8077125

[15] Bradley E, Green D. Involved, inputting or informing: “Shared” decision making in adult mental health care. Health Expect. 2018;21:192–200. 10.1111/hex.12601. 28779520 PMC5750775

[16] Zini MLL, Banfi G. A narrative literature review of bias in collecting patient reported outcomes measures (PROMs). Int J Environ Res Public Health. 2021;18:12445. 10.3390/ijerph182312445. 34886170 PMC8656687

[17] Awad AG. ‘The patient’: At the center of patient-reported outcomes. Expert Rev Pharmacoecon Outcomes Res. 2015;15:729–31. 10.1586/14737167.2015.1077118. 26289737

[18] Mendlovic S, Roe D, Markusfeld G, Mainz J, Kristensen S, Goldzweig G. Exploring the relation between clinician ratings and patient-reported experience and outcomes. Int J Qual Health Care. 2022;34:ii98–104. 10.1093/intqhc/mzac004. 35357441

[19] McIntyre RS, Ismail Z, Watling CP, Weiss C, Meehan SR, Musingarimi P, et al. Patient-reported outcome measures for life engagement in mental health: a systematic review. J Pat Rep Outc. 2022;6:62. 10.1186/s41687-022-00468-5. PMC9187792 35689159

[20] Hershenberg R, McDonald WM, Crowell A, Riva-Posse P, Craighead WE, Mayberg HS, et al. Concordance between clinician-rated and patient reported outcome measures of depressive symptoms in treatment resistant depression. J Affect Disord. 2020;266:22–9. 10.1016/j.jad.2020.01.108. 32056880 PMC8672917

[21] Scanferla E, de Bienassis K, Pachoud B, Gorwood P. How subjective well-being, patient-reported clinical improvement (PROMs) and experience of care (PREMs) relate in an acute psychiatric care setting? Eur Psychiatry. 2023;66:e26. 10.1192/j.eurpsy.2023.12. 36797203 PMC10044307

[22] Wolpert M, Curtis-Tyler K, Edbrooke-Childs J. A qualitative exploration of patient and clinician views on patient reported outcome measures in child mental health and diabetes services. Adm Policy Ment Health Ment Health Serv Res. 2016;43:309–15. 10.1007/s10488-014-0586-9. PMC4831988 25179754

[23] Boateng GO, Neilands TB, Frongillo EA, Melgar-Quiñonez HR, Young SL. Best practices for developing and validating scales for health, social, and behavioral research: a primer. Front Publ Health. 2018;6:149. 10.3389/fpubh.2018.00149. PMC6004510 29942800

[24] Buila SMD, Swanke JR. Patient-centered mental health care: encouraging caregiver participation. Care Manag J. 2010;11:146–50. 10.1891/1521-0987.11.3.146. 20839479

[25] McIntyre RS, Konarski JZ, Editorial GS. Sharpening the focus in mood disorders: from disease models to individualized measurement-based care. Ann Clin Psychiatry. 2007;19(4):213. 10.1080/10401230701653211. 18058278

