# Advancing measurement-based care through triangle of care: Development and feasibility of the Transdiagnostic Global Impression – Psychopathology scale for patients and informants – ADDENDUM

**DOI:** 10.1192/j.eurpsy.2025.10136

**Published:** 2025-11-20

**Authors:** Roger S. McIntyre, Zsofia Borbala Dombi, Agota Barabassy, Thomas Brevig, György Németh, Christoph U. Correll

**Keywords:** measurement-based care, psychiatric scale, psychopathology, transdiagnostic assessment tool, triangle of care

When this article was published online, two supplementary files were not uploaded. The publisher apologises for this omission.

The additional supplementary material has now been uploaded and can be found at https://doi.org/10.1192/j.eurpsy.2025.10069.
