# Supplementary material for: Advancing measurement-based care through triangle of care: Development and feasibility of the Transdiagnostic Global Impression – Psychopathology scale for patients and informants
Source: Eur Psychiatry. 2025 Aug 5;68(1):e118. doi: 10.1192/j.eurpsy.2025.10069 (PMC12438986; doi:10.1192/j.eurpsy.2025.10069)
Supplement: McIntyre et al. supplementary material [file S0924933825100692sup001.docx]

**Supplements**

*See attachments*

*Supplementary Table: TGI-P Patient version*

|  | **ORIGINAL WORDING** | **FINAL WORDING** |
| --- | --- | --- |
|  | In the past week, to what extent have you… | In the past week, 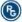how severe were your experiences of… |
| 1 | … experienced unusual thoughts or beliefs, seen or heard things that others did not, or had trouble organizing your thoughts or speech? | … having strong beliefs that others didn’t share, seeing or hearing things that others could not, or finding it difficult to think or speak clearly? |
| 2 | … felt being tense, irritable, angry, or easily annoyed, or noticed changes in how you react, like being more impulsive, uncooperative, or aggressive? | … feeling being tense, irritable, angry, or easily annoyed, or noticing changes in how you reacted, like being more impulsive or aggressive? |
| 3 | … felt unusually happy or confident, had racing thoughts, felt more energetic than usual, or got very involved in many fun activities? | … feeling unusually happy, confident, or energetic, having racing thoughts, or becoming involved in fun or exciting activities? |
| 4 | … felt unable to control your use of substances like tobacco, alcohol, or cannabis, had strong cravings, or felt you needed them to get through the day? | … being unable to control your use of substances like tobacco, alcohol, or cannabis — including experiencing strong cravings or feeling you needed them to get through the day? |
| **5** | … had trouble sleeping or felt excessively sleepy during the day? | … having trouble falling asleep or staying asleep, or waking up early? |
|  |  | … sleeping much more than usual or feeling very sleepy during the day? |
| 6 | … felt less interested in social activities, less motivated, or not enjoyed activities as much as before? | … feeling less motivated or interested in social activities, or not enjoying activities as much as before? |
| 7 | … felt like hurting yourself, had thoughts about life not being worth living, or about ending your life, or made any plans or attempts to do so? | … having thoughts about hurting yourself or ending your life and making any plans or attempts to do so? |
| 8 | … felt down, sad, or hopeless, or found it hard to enjoy things you used to like? | … feeling down, sad, or hopeless, or finding it difficult to enjoy things you used to like? |
| 9 | … had trouble concentrating, paying attention, remembering things or making decisions alone? | … having trouble concentrating, paying attention, remembering things or making decisions alone? |
| 10 | … anxiety made you feel restless, tense, panicky, or withdrawn from activities and social interactions? | … feeling nervous, restless, tense or panicky, or feeling unable to relax and needing to pace around? |
|  |  | … feeling constantly worried, or fearful of interacting with others and needed to withdraw from usual activities? |

*Supplementary Table: TGI-P Informant version*

|  | **ORIGINAL WORDING** | **FINAL WORDING** |
| --- | --- | --- |
|  | In the past week, how much have you noticed that the one you provide care for … | In the past week, how severe were the symptoms / experiences of the person you care for in terms of… |
| 1 | … experienced unusual thoughts or beliefs, seen or heard things that others did not, or had trouble organizing their thoughts or speech? | … having strong beliefs that others didn’t share, seeing or hearing things that others could not, or finding it difficult to think or speak clearly? |
| 2 | … felt tense, irritable, angry, or easily annoyed, or reacted differently, like being more impulsive, uncooperative, or aggressive? | … feeling being tense, irritable, angry, or easily annoyed, or noticing changes in how they reacted, like being more impulsive or aggressive? |
| 3 | … felt unusually happy or confident, had racing thoughts, felt more energetic than usual, or got very involved in many fun activities | … feeling unusually happy, confident, or energetic, having racing thoughts, or becoming involved in fun or exciting activities? |
| 4 | … felt unable to control their use of substances like tobacco, alcohol, or cannabis, had strong cravings, or felt they needed them to get through the day? | … being unable to control their use of substances like tobacco, alcohol, or cannabis — including experiencing strong cravings or feeling you needed them to get through the day? |
| **5** | … slept much more than usual or felt very sleepy during the day? | … having trouble falling asleep or staying asleep, or waking up early? |
|  |  | … sleeping much more than usual or feeling very sleepy during the day? |
| 6 | …felt less interested in social activities, less motivated, or not enjoyed activities as much as before? | … feeling less motivated or interested in social activities, or not enjoying activities as much as before? |
| 7 | … felt like hurting themselves, had thoughts about life not being worth living, or about ending their life, or made any plans or attempts to do so? | … having thoughts about hurting themselves or ending their life and making any plans or attempts to do so? |
| 8 | … felt down, sad, or hopeless, or found it hard to enjoy things they used to like? | … feeling down, sad, or hopeless, or finding it difficult to enjoy things they used to like? |
| 9 | … had trouble concentrating, paying attention, remembering things, or making decisions alone? | … having trouble concentrating, paying attention, remembering things or making decisions alone? |
| 10 | … felt so anxious that they were fearful of interacting with others, needed to withdraw from usual activities, and that their thoughts revolved mostly around their worries? | … feeling nervous, restless, tense or panicky, or feeling unable to relax and needing to pace around? |
|  |  | … feeling constantly worried, or fearful of interacting with others and needed to withdraw from usual activities? |
